# Remarkable Case of Malformation

**Published:** 1858-09

**Authors:** 


*Remarkable Case of Malformation.* Reported by Wm. A. Green, M. D., of Starkville, Ga.

Was called to Mrs. L., Monday, January 5th, 1858. Had been in labor with her second child. Nothing unusual occurred during gestation or parturition. She gave birth to a child, over the average size, which cried lustily, seeming to indicate every function was regularly and properly performed. Upon a close examination, the following deformities were found to exist:

The spine began a curvature at the superior third of the cervical vertebræ, in a direction towards the right hypochondrium, to the top of the sacrum. The concavity of this curvature was filled with two or three sac like appendages, containing, apparently, a fluid and gas, movable and compressible. "A want of the spinous processes of three or four contiguous vertebræ, is not a very uncommon species of monstrosity." "This constitutes *spina bifida.*" "There is, usually, a soft fluctuating tumor in the situation of the malformed bones, caused by water, contained within the sheath of the spinal marrow."—Vide Ramsbotham's Obstetrics. (Keating.) Appendix M. p. 622. Below, upon each side of the sacrum, were two appendages, resembling the mammæ of woman. In front, between the point where the umbilicus was attached, and the symphisis pubis, was a protrusion of intestines, within the peritoneal sac, reducible by pressure, but returning when removed. Immediately under this hernia, the urine trickled, continuously, from two or three small openings, which could not be entered by the smallest probe. Below this, and hanging pendent from the *middle of the symphisis pubis*, were the testicles, *perfectly* formed. There was no trace, nor any portion of the penis. Behind the symphisis pubis, in juxta-contact, and at the extreme *anterior* portion of the perinæum, was an anus, well formed, through which the fæces passed. About an inch and a-half behind this, at the point of the os coxcygis, was another anus, that, upon examination, proved to be imperforate—a cul de sac.

The face of the infant, when first born, was perfectly black, but is changing to a mulberry hue. Numerous marks are upon its body, such as are frequently seen upon children. Every other portion of the child seems perfectly and symmetrically developed. Its bowels are regular, it is healthy, and rapidly growing. The complete, entire, absence of the penis, or *any portion of it*—the unusual, unheard-of-positions of the anus, testicles and anomalous passage of the urine, are extremely remarkable and interesting. The bladder has no urethra, through which to pass its urine, so these apertures must come in direct contact with, and even enter the fundus of the bladder.—*Southern Medical and Surgical Journal.*

